# Non-Serotonergic Neurotoxicity by MDMA (Ecstasy) in Neurons Derived from Mouse P19 Embryonal Carcinoma Cells

**DOI:** 10.1371/journal.pone.0166750

**Published:** 2016-11-18

**Authors:** Dina Popova, Andréas Forsblad, Sanaz Hashemian, Stig O. P. Jacobsson

**Affiliations:** Department of Pharmacology and Clinical Neuroscience, Umeå University, Umeå, Sweden; Universidade de Sao Paulo Instituto de Quimica, BRAZIL

## Abstract

3,4-methylenedioxymethamphetamine (MDMA; ecstasy) is a commonly abused recreational drug that causes neurotoxic effects in both humans and animals. The mechanism behind MDMA-induced neurotoxicity is suggested to be species-dependent and needs to be further investigated on the cellular level. In this study, the effects of MDMA in neuronally differentiated P19 mouse embryonal carcinoma cells have been examined. MDMA produces a concentration-, time- and temperature-dependent toxicity in differentiated P19 neurons, as measured by intracellular MTT reduction and extracellular LDH activity assays. The P19-derived neurons express both the serotonin reuptake transporter (SERT), that is functionally active, and the serotonin metabolizing enzyme monoamine oxidase A (MAO-A). The involvement of these proteins in the MDMA-induced toxicity was investigated by a pharmacological approach. The MAO inhibitors clorgyline and deprenyl, and the SERT inhibitor fluoxetine, *per se* or in combination, were not able to mimic the toxic effects of MDMA in the P19-derived neurons or block the MDMA-induced cell toxicity. Oxidative stress has been implicated in MDMA-induced neurotoxicity, but pre-treatment with the antioxidants α-tocopherol or *N*-acetylcysteine did not reveal any protective effects in the P19 neurons. Involvement of mitochondria in the MDMA-induced cytotoxicity was also examined, but MDMA did not alter the mitochondrial membrane potential (ΔΨm) in the P19 neurons. We conclude that MDMA produce a concentration-, time- and temperature-dependent neurotoxicity and our results suggest that the mechanism behind MDMA-induced toxicity in mouse-derived neurons do not involve the serotonergic system, oxidative stress or mitochondrial dysfunction.

## Introduction

3,4-methylenedioxymethamphetamine (MDMA), colloquially known as ‘ecstasy’, is a ring-substituted phenylethylamine and a chemical derivative of amphetamine. Due to its psychostimulatory effects, MDMA is a popular recreational drug [1]. Repeated dosing or a single high-dose of MDMA can produce a variety of neurological disorders including cognitive impairments and mood disturbances [2], and MDMA is toxic to the nervous system.

The neurotoxic mechanism of MDMA is not fully understood. Metabolism of MDMA to neurotoxic metabolites [3], oxidative stress [4,5], glutamate excitotoxicity [6,7], mitochondrial dysfunction [8,9] and enhanced neurotoxicity secondary to a dose-dependent hyperthermia [10] have all been implicated. However, the main focus has been on the sympathomimetic properties of MDMA characteristic of phenylethylamine stimulants. Amphetamines, including MDMA, are amphiphilic compounds that cross the blood–brain barrier [11,12], and in the central nervous system (CNS) they are substrates for transporters of biogenic amines [13]. Neuronal accumulation of the amphetamines leads to elevated extracellular concentrations of monoamines. The cytoplasmic concentration of monoamines is also increased since amphetamines disrupt the vesicular uptake of neurotransmitters [14] and inhibit the MAO enzymes [15], making the monoamines more readily available for reverse transport into the synaptic cleft and, consequently, the monoamine levels in the synaptic cleft are even further increased.

Administration of MDMA to rodents produce long-lasting damage to serotonergic and dopaminergic neurotransmitter systems in the brain [16,17], including depletion of 5-hydoxytryptamine (5-HT; serotonin) [18] and dopamine (DA) [19], and a decrease in the density of serotonin transporters (SERT) [20], dopamine transporters (DAT) [17,21], as well as the rate-limiting enzymes of serotonin and dopamine biosynthesis (tryptophan hydroxylase [22] and tyrosine hydroxylase [17], respectively).

The pharmacology and toxicology of MDMA is species-dependent. In non-human primates and rats, MDMA produces a loss of nerve 5-HT terminals [16,23]. In mice, both the dopamine and the serotonin systems are affected depending on the mouse strain, brain region and dosage regimen studied. Repeated MDMA administration to mice decreases the DA content and leads to degeneration of nerve terminals in the striatum, leaving the 5-HT concentrations nearly intact [24,25]. In the frontal cortex and the hippocampus, MDMA decreases 5-HT concentrations, and additionally in the striatum, MDMA reduces the density of SERT [26]. MDMA has a high affinity for rodent SERT compared to other monoamine transporters [27,28], but in humans the affinity of MDMA is higher for the noradrenaline transporter (NET) [27,29]. These discrepancies highlight the importance of a deeper understanding of the mechanisms involved in MDMA-induced neurotoxicity. There are a large number of investigations on the neurotoxic effects of MDMA in rats, however less studies have been conducted in mice, especially in mouse-derived neuronal cell cultures.

In the mouse brain, the toxicity of MDMA on the dopamine system has been extensively investigated, and less attention has been paid to the effects on the serotonin system. One way of investigating the effects of MDMA on the serotonergic signaling is the use of *in vitro* models. Very little is known about the neurotoxicity of MDMA in neuronal cell cultures derived from mice [30,31]. In one of the few available studies [30], toxicity of ecstasy (MDMA) towards embryonic stem cell-derived cardiac and neural cells showed that MDMA had toxic effects upon cardiomyocytes and neurons derived from mouse embryonic stem cells. Interestingly, in this study, MDMA was suggested to have more potent toxicity on the neural differentiation process rather than the cardiac differentiation. There is thus clearly a need to investigate further the toxicity of MDMA in mouse-derived neuronal cultures.

Mouse P19 embryonal carcinoma (EC)-derived neurons are a useful model with predictive validity for screening of drug- and chemical-induced neurotoxicity [32]. The P19 EC cells, isolated from a teratocarcinoma in C3H/He mice [33], are pluripotent and can differentiate in culture into many tissue types similar to those normally found in early embryos. P19 cells resemble those of the inner mass of the blastocyst, and their differentiation is believed to closely mimic critical events in early embryogenesis. Retinoic acid (RA)-treated P19 cells serves as an *in vitro* model system to study early steps in neuronal development, since RA effectively induces the development of neurons, astroglia and microglia, cell types normally derived from the neuroectoderm [34]. RA-induced P19-derived neurons show fundamental phenotypes of neurons in the mammalian nervous system, including irreversibly postmitotic cells, functional inhibitory and excitatory synapses, and expression of a number of different neurotransmitters and their cognate receptors [35–42]. In the present study, we have used P19-derived neurons to investigate the neurotoxic properties of MDMA *in vitro*, and to determine whether or not the serotoninergic system plays a part in the toxicity.

## Materials and Methods

### Chemicals

MEM-α medium containing deoxyribonucleosides and ribonucleosides, MEM medium with Earle’s salts and L-glutamine, fetal bovine serum (FBS), penicillin-streptomycin (PEST), MEM non-essential amino acids (NEAA), Neurobasal medium, B27 supplement, L-glutamine and Hank’s balanced salt solution (HBSS) with CaCl_2_ and MgCl_2_ were purchased from Invitrogen Life Technologies (Uppsala, Sweden). All-trans retinoic acid, poly-D-lysine hydrobromide, (*±)*-3,4-methylenedioxymethamphetamine hydrochloride (MDMA), serotonin hydrochloride, ketanserin (+)-tartrate salt, *N*-acetyl-L-cysteine (NAC), (+)- α-tocopherol, R-(-)- deprenyl hydrochloride, *N*-methyl-*N*-propargyl-3-(2,4-dichlorophenoxy) propylamine hydrochloride (clorgyline), clomipramine hydrochloride, fluoxetine hydrochloride, citalopram hydrobromide, pargyline hydrochloride, dimethyl sulfoxid (DMSO), thiazolyl blue tetrazolium bromide (MTT), bovine serum albumin and RIPA buffer were purchased from Sigma-Aldrich (Stockholm, Sweden). Cytotoxicity detection kit (LDH) was obtained from Roche Diagnostics (Mannheim, Germany). Protease inhibitor cocktail set III was obtained from EMD Millipore Corp. (Billerica, MA, USA). Pierce^®^ BCA protein assay kit was purchased from Thermo Scientific (Rockford, IL, USA). PROTEAN^®^ TGX^™^ Precast gels, 0.2 μm PVDF Trans-Blot^®^ Turbo^™^ transfer pack, Clarity^™^ Western ECL Substrate were obtained from BIO-RAD Laboratories, Inc. (USA). Anti-monoamine oxidase A antibody [EPR7101] (ab126751) and TMRE mitochondrial membrane potential assay kit were purchased from Abcam (Cambridge, UK). Polyclonal goat anti-rabbit immunoglobulins/HRP was obtained from Dako (Glostrup, Denmark).

RNeasy mini kit, miRNeasy mini kit, Taq PCR Core kit were purchased from QIAGEN (Sollentuna, Sweden). High capacity cDNA reverse transcription kit was obtained from Applied Biosystems (Stockholm, Sweden). GelRed nucleic acid gel stain was purchased from Biotium (Hayward, CA, USA). Agarose standard was obtained from Saveen Werner AB (Limhamn, Sweden). mRNA extraction Dynabeads^®^ Direct^™^ kit was obtained from Ambion, Life Technologies AS (Oslo, Norway). KAPA SYBR^®^ FAST qPCR Master Mix was purchased from KAPA BIOSYSTEMS Ltd. (London, UK).

ELISA kit for serotonin transporter (SERT) (mus musculus) was purchased from USCN Life Science Inc. (Hubei, P.R. China). Hydroxytryptamine, 5-[1,2-^3^H] creatinine sulfate was obtained from American Radiolabeled Chemicals, Inc. (St Louis, MO, USA).

### Cell culture

P19 cells (passage 18–34) from European Collection of Cell Cultures (Porton Down, U.K.) were grown in T75 flasks in MEM-α medium supplemented with 10% FBS, 100 units/ml penicillin-streptomycin (PEST) and 1% MEM non-essential amino acids (NEAA). Cells were kept at 37°C in an incubator with humidified atmosphere and supplied with 5% CO_2_. Cells were passaged every fourth day at 70–80% of confluence. P19 cells were induced to neuronal differentiation essentially as described in Yao et al. (1995) [43] and cultured in Neurobasal medium with B27 supplement according to Svensson et al. (2006) [42].

Briefly, the process of neuronal differentiation was induced by plating 1 × 10^6^ cells in MEM-α medium (5% FBS, 1% PEST and 1% NEAA) containing 1 μM all-trans retinoic acid (RA) for 4 days on bacterial-grade Petri dishes (Ø 92 mm; Sarstedt Inc., Newton, NC) leading to the formation of cell aggregates. The medium was replaced after 48 h. After a total of 96-hours of exposure to RA, the aggregates were trypsinized for 10 min, dissociated and plated in Neurobasal medium with 2% B27 serum-free supplement, 1 mM L-glutamine and 1% PEST, into poly-D-lysine pre-coated (50 μg/ml) 96-well, 12-well or 6-well culture plates at a density of 500–1000 cells/mm^2^. Half of the medium per well was replaced every 48 h. Experiments were conducted 7–10 days after plating the cells in the serum-free media.

Undifferentiated P19 cells, for the experiments, were grown overnight in 6-well culture plates at the density of 500–750 cells/mm^2^ in the culturing medium containing 1% FBS.

HepG2 cells (Passage 108–128) obtained from European Collection of Cell Cultures (Porton Down, U.K.) were cultured in MEM medium containing 10% FBS, 100 units/ml penicillin-streptomycin (PEST) in T75 flasks. When reached 70–80% of confluence, the cells were split 1:3 or 1:4.

### Neurotoxicity assays

All test substances, except fluoxetine and α-tocopherol, were dissolved in the cell culture medium. Fluoxetine was dissolved in DMSO and α-tocopherol in ethanol with the final vehicle concentrations set to 0.1% and 0.5%, respectively. Fluoxetine was added to the cells 30 min prior to MDMA exposure.

The cell membrane integrity was investigated by measuring LDH activity in culture medium. Aliquots (100 μl/well) were transferred to an optically clear 96-well flat bottom microtiter plate followed by the addition of 100 μl of the Cytotoxicity Detection Kit assay mixture. After 30 min of incubation at room temperature, the samples were measured spectrophotometrically at 490 nm (reference wavelength 650 nm) in the SPECTROstar Nano absorbance microplate reader (BMG LABTECH GmbH, Offenburg, Germany). To determine the total LDH content, aliquots from wells incubated with 2% Triton X-100 solution for 30 min, 37°C, 5% CO_2_ were used.

Cell viability was measured with MTT reduction assay [44]. After taking 100 μl of the medium for determination of LDH activity as described above, 10 μl of 5 mg/ml MTT dissolved in PBS, pH 7.2 was added to the wells. During a three hour incubation at 37°C, 5% CO_2_, viable cells formed purple formazan crystals, that were dissolved by adding 100 μl of 0.01 M HCl in 10% SDS. The plates were incubated overnight at room temperature and measured spectrophotometrically at 570 nm with a reference wavelength of 650 nm in the SPECTROstar Nano microplate reader.

### Mitochondrial membrane potential (ΔΨm) analysis

Mitochondrial membrane potential was measured with a TMRE (tetramethylrhodamine ethyl ester) assay. P19 neurons (750 cells/mm^2^) were exposed to MDMA on days 7–9 in serum-free medium for 10 min up to 48 hours. The positive control FCCP (carbonyl cyanide-*p*-trifluoromethoxyphenylhydrazone), an uncoupler of mitochondrial oxidative phosphorylation, was applied at the concentration of 5 μM for 10 min. The cells were incubated with 500 μM TMRE for 30–45 min at 37°C, 5% CO_2_, followed by washing once with 100 μl of HBSS containing 0.2% bovine serum albumin. A volume of 200 μl of HBSS containing 0.2% bovine serum albumin was added to each well, and the fluorescence was measured in the FLUOstar Galaxy plate reader (BMG Labtechnologies GmbH, Offenburg, Germany) with excitation/emission: 544/590 nm.

### SERT expression analyses

#### Reverse transcription PCR

Total RNA was extracted from RA-differentiated (at day 8 in the serum-free media) and undifferentiated P19 cells using RNeasy mini kit and from the C57BL/6 mouse brain (cerebrum) using miRNeasy mini Kit according to the manufacturer’s instructions. The RNA was quantified with NanoDrop Lite spectrophotometer (ThermoFisher Scientific, Shanghai, P.R. China) and reverse-transcribed to cDNA using High Capacity cDNA Reverse Transcription kit. The cDNA template (4 ng/reaction) was used in the end-point PCR analyses. SERT fragment (127 bp) was amplified with the primers 5´-TGCCTTTTATATCGCCTCCTAC-3´(forward) and 5´-CAGTTGCCAGTGTTCCAAGA-3´ (reverse) according to the PCR program: 3 min at 94°C followed by 35 cycles each of 45 s at 94°C, 45 s at 60°C and 60 s at 72°C. PCR products were analyzed with agarose gel electrophoresis on 1.2% agarose gel stained with GelRed.

#### Real-time quantitative PCR

For mRNA extraction, P19 cells and RA-differentiated P19 cells (at days 8 and 10 in the serum-free medium) plated in 6-well plates at the density of 750 cells/mm^2^ were washed with PBS, lysed with 600 μl/well of the Lysis buffer (Dynabeads^®^ mRNA Direct kit) and stored at -80°C. mRNA was extracted with Dynabeads^®^ mRNA Direct kit according to the manufacturer’s instructions. cDNA was synthesized using High Capacity cDNA Reverse Transcription kit from 50 ng of mRNA. Quantitative PCR (qPCR) was performed with Eco^™^ instrument and software (Illumina, Inc., San Diego, CA, USA). PCR reactions were run with 1.6 μl of cDNA in a total volume of 20 μl using a SYBR Green mix (KAPA SYBR^®^ FAST qPCR Master Mix). Each sample was run in duplicate. The conditions used for amplification were: 10 min at 95°C, followed by 45 cycles of 10 s at 95°C, 30 s at 60°C and 15 s at 72°C. A°C melting curve was performed at the end of the PCR reaction to analyze the products. Data were normalized to the 60S ribosomal protein L19 (RPL19) mRNA expression. Primer sequences for SERT: 5´-GCTGATGATGTAAGGTCTTTCTCC-3´(forward) and 5´-AGTCCAAGAGAGTTCATGGAAAG-3´ (reverse), and for RPL19: 5´-TACTGCCAACGCTCGCAT-3´ (forward) and 5´-AACACATTCCCTTTGACCTTCA-3´(reverse).

#### ELISA for serotonin transporter

The presence of serotonin transporters in neuronally differentiated and undifferentiated P19 cells was determined with an ELISA kit for serotonin transporter (SERT) (mus musculus) according to the manufacturer’s instructions with some modifications. To obtain cell lysates, the cells were washed three times with ice-cold PBS, scraped and centrifuged for 5 min, ~200 G, 4°C in Allegra 25R Centrifuge, Beckman Coulter, Palo Alto, CA, USA. The pellets were diluted in 0.5 ml PBS and sonicated in Branson sonifier cell disruptor B15 (Branson Sonic power company, Heusenstamm, Germany) with five pulses five times (30 watt power output, 50% duty cycle). The cell homogenates were centrifuged at 1500 G for 10 min at 4°C and the supernatants were stored at -80°C until used for the assay. The protein concentrations were determined with Pierce BCA protein assay kit (Thermo Scientific, Rockford, IL, USA). For the ELISA assay, the proteins were used at the concentration of 2.5 mg/ml, and 100 μl of standard, blank and samples were added to the microtiter plate pre-coated with an antibody specific to SERT and incubated for 2 h at 37°C. The liquid was removed and 100 μl/well of the biotin-conjugated detection antibody specific to SERT was added for 1 h incubation at 37°C. The plate was then washed three times with 350 μl washing buffer per well and 100 μl of the avidin conjugated to horseradish peroxidase (HRP) was added to each well followed by 30 min incubation at 37°C. The plate was washed five times with the washing buffer and 90 μl/well of the TMB substrate solution was added followed by incubation for 22 min at 37°C. The reaction was stopped by adding 50 μl of the stop solution per well and the absorbance was measured at 450 nm in the SPECTROstar Nano microplate reader (BMG LABTECH GmbH, Offenburg, Germany).

#### Serotonin uptake

Serotonin uptake was measured in RA-differentiated P19 cells essentially as described by Rudd et al. (2005) [45] with the following modifications. RA-induced cells were plated at the density of 1000 cells/mm^2^ in 12-well plates and allowed to differentiate for eight days in the serum-free medium. A plate containing medium only was used in each experiment to determine the non-specific retention of [^3^H]-5-HT in the wells.

Culture plates with (or without) cells were washed twice with 1 ml uptake buffer (140 mM NaCl, 2 mM KCl, 1 mM CaCl_2_, 1 mM MgCl_2_, 5 mM d-glucose, 10 mM HEPES, pH 7.4). Citalopram (1 μM), MDMA (1 mM) or the vehicle DMSO (0.002%) were added for a 10 min incubation at 37°C in the uptake buffer containing 250 μM ascorbic acid, 10 μM pargyline and 0.1% fatty acid free bovine serum albumin. Serotonin (100 nM) and [^3^H]-5-HT (1.5 nM) were added to the wells and allowed to incubate for 30 min at 37°C. The uptake process was terminated by washing the cells two times with 1 ml ice-cold uptake buffer. The cells were lysed in 1 ml 0.2 M NaOH for 15 min in 75°C and 0.5 ml of the lysate samples were transferred to the scintillation vials. Tritium content was determined by liquid scintillation spectroscopy with quench correction.

### Western blotting

For collection of the cell lysates, the cells were washed twice in PBS, dislodged by scratching in 5 ml PBS and transferred to 15 ml Falcon conical tubes. After centrifugation (5 min at ~200 G, 4°C), the pellets were lysed with RIPA buffer containing protease inhibitor cocktail III (1:200), constantly agitated for 30 min, 4°C, sonicated in a Branson sonifier cell distruptor B15 (Branson Sonic power company, Heusenstamm, Germany) for 5 sec (30 watt power output, 50% duty cycle) and centrifuged for 5 min, 14000 G, 4°C. The supernatants were stored at -80°C. The protein concentrations were determined with Pierce^®^ BCA protein assay kit. The proteins (10 μg) were separated by SDS-PAGE using Mini-Protean^®^ Tetra System on Mini-PROTEAN^®^ TGX^™^ Precast gels and transferred on PVDF membranes in Trans-Blot^®^ Turbo^™^ transfer system (7 min, 18 V) (BIO-RAD Laboratories, Inc., USA). The membranes were incubated with rabbit monoclonal anti-monoamine oxidase A antibodies overnight at 4°C. HRP conjugated polyclonal goat anti-rabbit secondary antibodies were applied for 1 h at room temperature. Antibodies bound to proteins were detected with chemiluminiscence using Clarity^™^ Western ECL Substrate and the images were captured with Image Lab^™^ Software (BIO-RAD Laboratories, Inc., USA).

### Statistical analysis

Statistical analyses (one- or two-way ANOVA for repeated measures with Dunnett’s or Bonferroni's post hoc multiple comparisons tests) were undertaken in the GraphPad Prism computer program (GraphPad Software Inc., San Diego, CA, USA).

## Results

### Expression of SERT and MAO-A in P19 cells and P19-derived neurons

**Fig 1:**
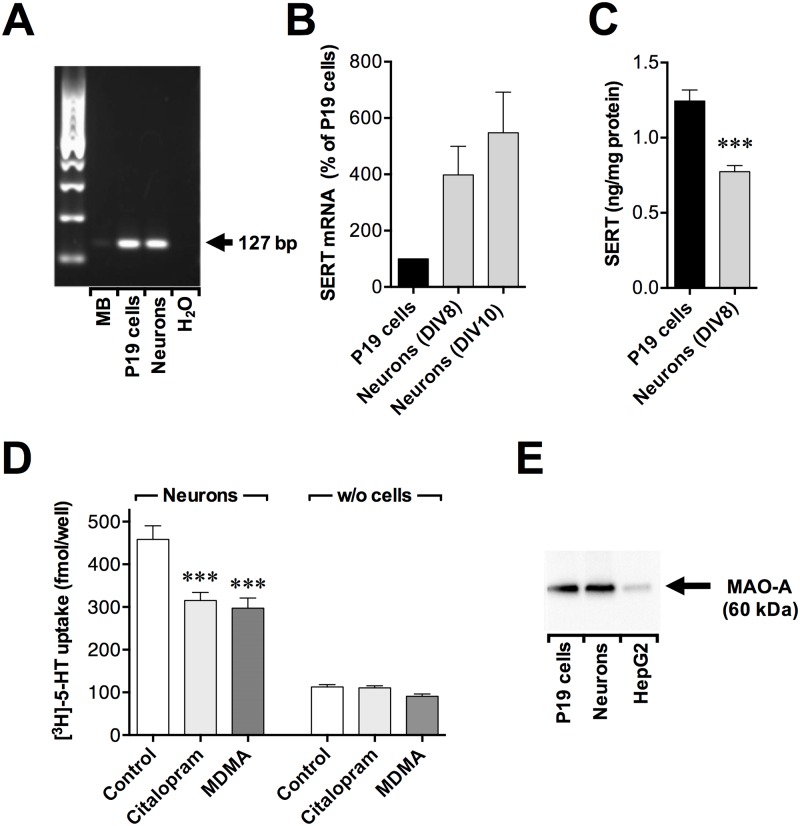
Expression of serotonin transporter and monoamine oxidase A in P19 cells and P19 neurons. (A) Reverse transcription PCR analysis of the mRNA expressions of SERT in P19 cells and P19 neurons (at day 8 in the serum-free media) with a mouse brain lysate (MB) as a positive control. Total RNA was isolated and reverse transcribed into cDNA. The PCR products were analysed by agarose gel electrophoresis and fragment size estimated using a 100 bp marker. The arrow shows the expected amplicon size for SERT (127 bp) with the primer pair used. (B) qPCR analysis of mRNA expression levels of SERT in P19 cells and neuronally differentiated P19 cells (at days 8 and 10 in the serum-free media) with RPL19 as housekeeping gene. Results are expressed as a percentage of P19 cells. Values are means ± SEM of n = 5 independent experiments. (C) Expression of SERT in P19 cells and P19 neurons (at day 8 in the serum-free media) as measured with ELISA. Data are means ± SEM of n = 4 independent cell preparations (P19 neurons) and 5 (P19 cells). Statistical analysis (unpaired t test) showed a significant difference between P19 cells and neurons (***p< 0.001). (D) Effects of the selective SERT inhibitor citalopram and MDMA on 5-HT uptake in P19 neurons (at day 8 in the serum-free media). The cells (or wells without cells) were preincubated for 10 min with 1 μM citalopram, 1 mM MDMA or 0.002% DMSO as vehicle control followed by 30 min incubation with 100 nM [^3^H]-5-HT at 37°C. Data are means ± SEM of n = 8 independent experiments. Statistical analysis was performed using one-way repeated measures ANOVA with post hoc Bonferroni’s multiple comparison test (***p<0.001 for citalopram- and MDMA- vs. vehicle-treated control cells, no statistically significant difference was observed between citalopram-treated and MDMA-treated cells). (E) Western blot analysis with rabbit anti-monoamine oxidase A monoclonal antibody (ab126751) (Abcam). Comparison of immunoreactivity between P19 cells, P19 neurons (at day 10 in the serum-free media) and the positive control human liver hepatocellular carcinoma cell line (HepG2). Cell lysates: 10 μg per lane. The arrow shows the expected size of MAO-A (60 kDA).

SERT was expressed on the mRNA level (Fig 1A and 1B) and on the protein level (Fig 1C) in both P19 cells and RA-induced P19 neurons. The mRNA levels of SERT were higher in P19 neurons (at days 8 and 10 in the serum-free media) compare to P19 cells (Fig 1B). The protein levels of SERT were, however, moderately lower in P19-derived neurons (at day 8 in the serum-free media) compare to undifferentiated P19 cells (Fig 1C). The serotonin uptake detected in the RA-induced P19 neurons was citalopram-sensitive, and attenuated by 1 mM MDMA (Fig 1D). The protein monoamine oxidase A was detected in both P19 cells and P19 neurons (at day 10 in the serum-free medium) with Western Blot (Fig 1E). Taken together, these data suggest that the P19 neurons express known targets for MDMA, including a functional serotonin reuptake transporter.

### Effects of MDMA on cell viability

**Fig 2:**
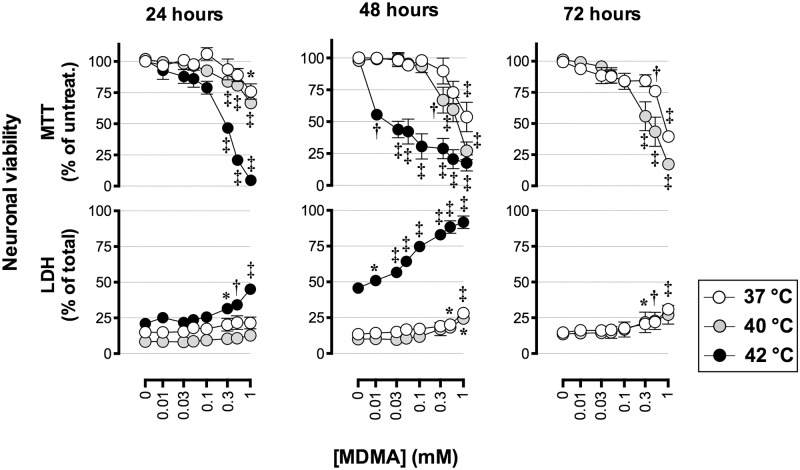
Concentration-, time- and temperature- dependent effects of MDMA on the viability of P19 neurons. MTT reduction and LDH release were measured in P19-derived neurons exposed to MDMA at day 7 in the serum-free media for 24 h, 48 h and 72 h at the temperatures 37°C, 40°C and 42°C, in a humified atmosphere with 5% CO_2_. Data are means ± SEM of n = 4–5 independent experiments. For MTT reduction, data are expressed as percentage of non-treated control cells. For LDH release, the results are presented as percentage of total cell death (cells treated with 2% Triton X-100). Statistical analysis was performed using one-way ANOVA with post hoc Dunnett’s multiple comparisons test (*p< 0.05, †p< 0.01, ‡p< 0.001) compared to corresponding controls.

The neurotoxic effect of MDMA was examined at normothermic (37°C) and hyperthermic conditions (40°C and 42°C) since ambient temperatures significantly affected the neurotoxicity produced by MDMA in laboratory animals and cultured primary rat cortical neurons [46, 47]. MDMA produced a concentration-, time-, and temperature- dependent toxicity in P19 neurons treated on day 7 in the serum-free-medium (Fig 2). After 24, 48 and 72 h of exposure to 1 mM MDMA at 37°C, cellular MTT reduction was 76% (±6.3), 54% (±11.6) and 40% (±4.1) (means ± SEM) of untreated cells, respectively. Under the same conditions, LDH release after 48 h was 28% (± 3.1) of total LDH release (compare to 13% ± 1.2 in untreated controls) and after 72 h 31% (±1.9) of total LDH release (compare to 16% ± 1.3 in untreated controls). At the hyperthermic condition of 40°C, MTT reduction in P19 neurons exposed to 1 mM MDMA for 24 h was 67% (±3.7), and at 42°C 5% (±1.8) of non-treated controls. Under the same conditions at 42°C, the extracellular content of LDH significantly increased to 45% (±2.7) of total LDH release compare to 21% ±1.6 in untreated controls.

**Fig 3:**
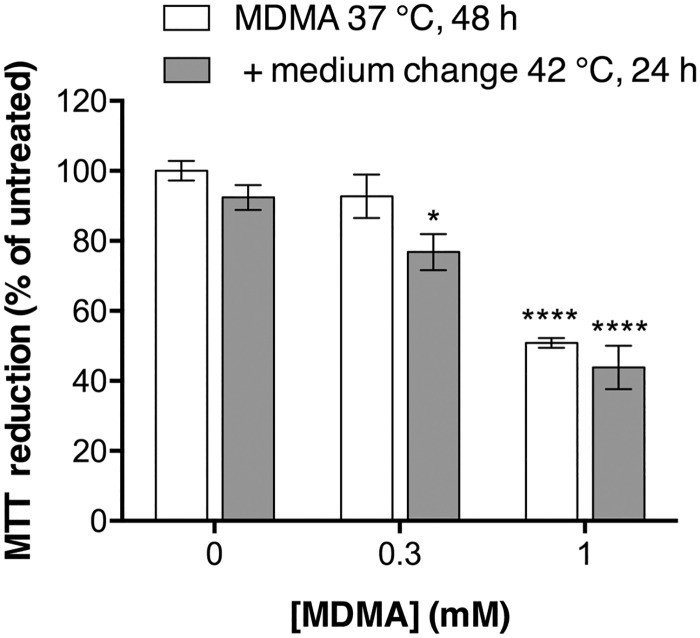
Effects of MDMA treatment prior to high temperature upon the MTT reduction produced by P19 neurons. P19 neurons were treated with MDMA on day 7 in the serum-free media at 37°C, 5% CO_2_ for 48 h without (white bars) or with (grey bars) an additional step whereby medium was changed to remove MDMA and the cells were incubated for a further 24 h at 42°C, in a humified atmosphere with 5% CO_2_. Cell viability was assessed with MTT reduction assay. Data (means ± SEM of n = 4 independent experiments) are presented as percentage of non-treated control cells. Statistical analysis was performed using two-way repeated-measures ANOVA matching both treatment and temperature. The interaction term treatment x temperature was not significant. The significant difference within temperature groups were followed up with post hoc Dunnett’s multiple comparison test compared to non-treated control cells (*p< 0.05, ****p< 0.0001).

The temperature-dependency shown in Fig 2 could reflect an ability of MDMA to sensitize the cells so that they become less resilient to high temperatures. In order to investigate this possibility, P19 neurons were exposed to 300 μM or 1 mM MDMA for 48 h at 37°C and the MTT reduction was measured. In parallel wells, the medium was then changed to remove MDMA and the cells were incubated for an additional 24 h at 42°C after which cell viability was assessed with the MTT reduction assay. If the MDMA had sensitised the cells to deleterious effects of a high temperature, then the cells with the additional incubation at 42°C would be expected to show lower rates of MTT reduction than those treated with MDMA and then assayed immediately. No such sensitization was observed (Fig 3).

### Comparison between MDMA and inhibitors of SERT and MAO in P19 neurons

Since MDMA has been shown to act by binding to SERT and MAO, we examined if the MAO-A inhibitor clorgyline, the MAO-B inhibitor deprenyl, and the SERT inhibitor fluoxetine, *per se* or in combination, could mimic the toxic effects of MDMA in P19 neurons (Table 1). As expected, MDMA reduced MTT in a concentration- and temperature-dependent manner, with the largest effect (to 33% of control) being seen following 24 h of incubation at 42°C. In contrast, neither clorgyline (1 μM), deprenyl (1 μM), fluoxetine (1 μM) or the combination of these compounds significantly affected the MTT reduction (Table 1).

**Table 1:** Effects of MDMA, clorgyline, deprenyl, fluoxetine and the combinations on MTT reduction in P19 neurons.

Compounds	24 hours of incubation			48 hours of incubation		
	37°C	40°C	42°C	37°C	40°C	42°C
Controls	98±2.7	95±3.1	104±2.5	100±2.8	97±5.6	105±4.6
0.1% DMSO	103±2.5	100±7.9	104±7.7	112±3.4	107±6.5	105±3.0
0.3 mM MDMA	90±9.0	86±5.0	71±4.3***	93±6.2	86±6.4	73±8.3*
1 mM MDMA	66±4.6**	61±4.4**	33±6.1****	51±1.4****	67±3.7*	42±13***
1 μM Clorg.	95±8.0	90±7.0	104±3.3	108±3.7	101±7.1	105±8.1
1 μM Depr.	110±3.6	103±6.2	112±5.6	112±7.9	113±3.8	113±6.2
1 μM Clorg.+ 1 μM Depr.	103±7.5	104±8.9	106±4.3	103±5.9	101±12	112±4.3
10 nM Fluox.	100±10	99±14	83±16	91±11	108±4.1	102±13
100 nM Fluox.	92±13	94±9.9	73±16	87±9.7	94±10	85±11
1 μM Fluox.	106±7.2	101±11	82±9.7	91±1.8	105±5.3	91±9.7
1 μM Clorg. + 1 μM Depr. + 1 μM Fluox.	96±7.7	94±9.1	76±6.9	82±8.8	86±5.4	98±13

The compounds were added to the cells on day 7 in the serum-free medium followed by 24 h and 48 h incubation at 37°C, 40°C and 42°C, in a humified atmosphere with 5% CO_2_. Data (means ± SEM of four independent experiments) are expressed as percent of untreated controls or DMSO vehicle for fluoxetine. Statistical analysis was performed using one-way ANOVA with post hoc Dunnett’s multiple comparisons test against untreated controls or DMSO for fluoxetine (*p<0.05, **p<0.01, ***p<0.001, ****p<0.0001).

Higher concentrations of fluoxetine, i.e. well above concentrations needed for blockade of SERT, were toxic to the P19 neurons, albeit in a manner that was not additive with MDMA (S1 Fig). Another compound capable of interacting with SERT, clomipramine, was also toxic to the P19 neurons at high concentrations (S2 Fig).

**Fig 4:**
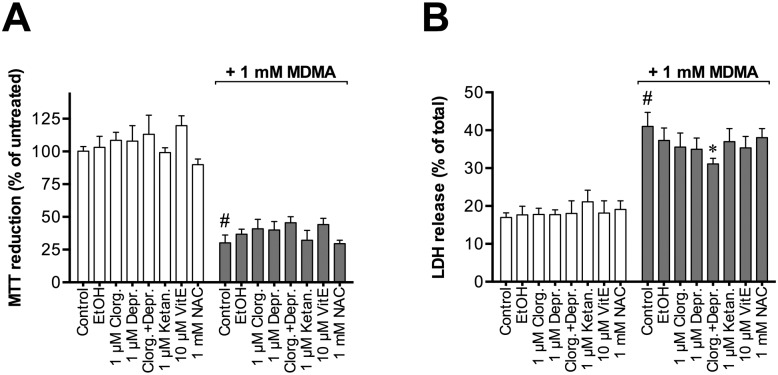
Effects of clorgyline, deprenyl, ketanserin, α-tocopherol (VitE) and N-acetylcysteine (NAC) on MDMA-induced toxicity in P19 neurons. P19 neurons were cultured for eight days in the serum-free media and incubated for 48 h with the test compounds in absence or presence of 1 mM MDMA. Cell viability was measured with (A) MTT reduction assay and (B) LDH activity assay. Data are means ± SEM of n = 3–4 independent experiments. For MTT reduction, data are expressed as percentage of non-treated control cells. For LDH release, the results are presented as percentage of total cell death (cells treated with 2% Triton X-100). Statistical analysis was performed using repeated measures one-way ANOVA with post hoc Bonferroni's multiple comparisons test (#p< 0.0001 for comparison between untreated control cells and the treatments, and *p< 0.05 when treatments in presence of MDMA are compared to 1 mM MDMA).

Further experiments indicated that a 48 hours of incubation of the P19 neurons with either the MAO inhibitors, the 5-HT_2A_ receptor antagonist ketanserin, or the antioxidants α-tocopherol and *N*-acetylcysteine (NAC) did not significantly affect either MTT reduction *per se* or affect the action of 1 mM MDMA upon this biochemical measure (Fig 4).

### No effects of MDMA on mitochondrial membrane potential

**Fig 5:**
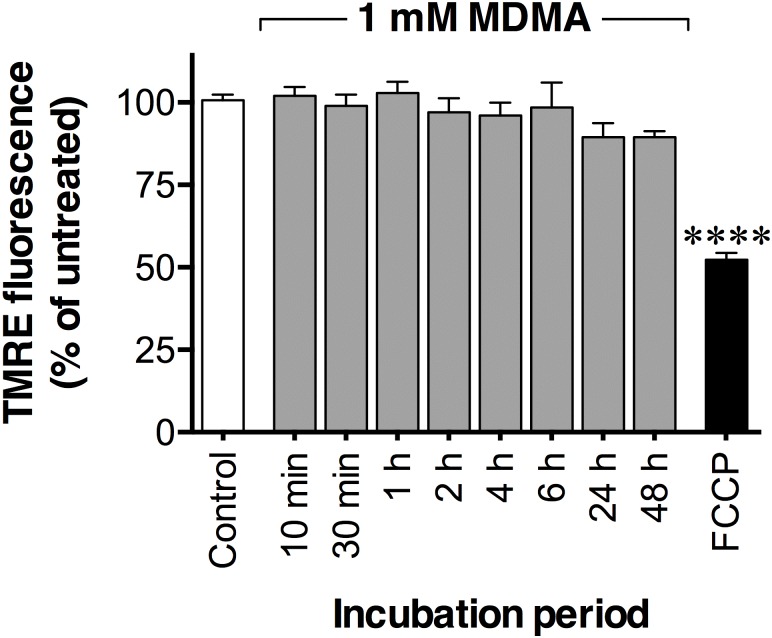
Time-dependent effects of MDMA on mitochondrial membrane potential. P19 neurons, cultured for seven to nine days in serum-free medium, were exposed to 1 mM MDMA for 10 min up to 48 h. Mitochondrial membrane potential was measured by using the TMRE assay. The mitochondrial oxidative phosphorylation uncoupler FCCP (5 μM) applied for 10 min was used as a positive control. Data are means ± SEM of n = 4–6 independent experiments. Statistical analysis was performed using one-way ANOVA with post hoc Dunnett’s multiple comparisons test (****p< 0.0001) compared to the untreated control.

Exposure of MDMA (up to 1 mM) for 10 min and up to 48 h did not alter the mitochondrial membrane potential in the P19 neurons as assessed with the TMRE assay (Fig 5).

## Discussion

In the present study, we have investigated the effects of MDMA on cell viability in neuronally differentiated mouse embryonal carcinoma P19 cells. The key findings of the present study include 1) that the toxicity produced by MDMA in P19 neurons was time- and temperature-dependent, and 2) that the mechanisms of toxicity did not involve inhibition of monoamine oxidase and/or the serotonin-re-uptake transporter, activation of 5-HT_2A_ receptors, oxidative stress or mitochondrial dysfunction.

P19-derived neurons are useful for neurotoxicity studies, and the cell model has been used to evaluate neurotoxic effects of compounds in a number of studies [32,48]. RA-treated P19 cells morphologically resemble cultured mammalian brain cells, they are post mitotic and have functional synapses [35,36]. Cholinergic and GABAergic properties were found in RA-differentiated P19 cells [35,49] and also enzymes for catecholamine synthesis [50]. In this study we additionally detected the presence of serotonin-re-uptake transporter and monoamine oxidase A in both P19 cells and P19 neurons. The levels of SERT mRNA were higher in P19 neurons than in P19 cells as detected with qRT-PCR. However, P19 neurons contained less of SERT protein than P19 cells as assessed with ELISA. The correlation between mRNA levels and protein abundance has been found weak but still positive in a number of studies [51] unlike the present results. Nevertheless, a functional serotonin reuptake transport was detected in the P19 neurons, that was attenuated by the selective serotonin reuptake inhibitor citalopram as well as by MDMA.

We have investigated the MDMA-toxicity in P19 neurons at normothermic (37°C) and hyperthermic conditions (40°C and 42°C). One of the most pronounced acute effects upon MDMA-intoxication in both humans [52] and experimental animals [53,54] is hyperthermia. In laboratory animals and in cultured primary rat cortical neurons, ambient temperatures significantly affected neurotoxic effects produced by MDMA [46,47]. In P19 neurons, MDMA produced toxicity in a temperature-, time- and concentration-dependent manner. However, MDMA did not sensitize the cells to deleterious effects of high temperature. Although the concentrations of MDMA were high (up to 1 mM) they corresponded to the concentrations required to obtain cytotoxicity in other studies using other cell culture systems [3,55,56]. In our mouse cell model, exposure to 1 mM MDMA at 37°C for 72 hours produced a maximal cell death of 60% (as shown by the MTT data). The lack of maximal toxicity (100%) could be due to factors such as degradation of MDMA, receptor desensitization, or proliferation of non-neuronal and/or MDMA-susceptible cells in the mouse P19 neuronal cultures. The model contains mainly neurons, although glial cells and a population of fibroblast-like cells have also been identified [34], but we have previously shown that there is no substantial proliferation of non-neuronal cells under the serum-free conditions employed in this study [32].

An established mechanism of MDMA- induced toxicity in the brain is an increase of extracellular 5-HT and DA levels via action on SERT and DAT both *in vitro* [57–60] and *in vivo* [61] with higher affinity binding to SERT [27]. MDMA also inhibits the enzyme monoamine oxidase [15] responsible for inactivation of serotonin and dopamine [62] that contributes to their elevated levels. Those actions of MDMA lead to persistent structural and functional damage of serotonergic nerve terminals in the brain [16,63].

For that reason, we investigated if we could mimic the toxic effects of MDMA in P19 neurons with the monoamine oxidase inhibitors clorgyline [64], deprenyl [64,65] and the SERT inhibitor fluoxetine [66] at different temperatures. However, in contrast to MDMA, these compounds and their combinations were not toxic to the cells, arguing against effects on these proteins as the cause of MDMA toxicity in the P19 neurons.

Pre-treatment with deprenyl, has been shown reduce reduce the serotonergic neurotoxicity produced by MDMA in the rat striatum [67] and protected rat brain mitochondria from MDMA-induced oxidative damage [68]. Pre-treatment with clorgyline, potentiated MDMA-induced increase in extracellular serotonin produced by MDMA in rat substantia nigra *in vivo* [69]. Clorgyline has also been shown to produce a synergistic effect on serotonin-mediated behaviour, body temperature and increased mortality in rats [9]. The inhibition of MAO-A by clorgyline did not protect rat brain mitochondria from oxidative stress produced by MDMA [9]. In our P19 neuronal model, neither of the monoamine oxidase inhibitors nor their combination had an effect on MDMA cytotoxicity confirming that monoamine oxidase was probably not involved in MDMA-induced toxicity in our model.

We also examined if fluoxetine could protect the P19 neurons against MDMA-induced toxicity since the protective effects of fluoxetine to serotonergic neurotoxicity were observed in the brain [70,71]. However, the compound did not have protective effects in our cell model. The fact that clomipramine, a tricyclic antidepressant that interacts with monoamine transporters [72], also showed neurotoxic effects in our P19 neuronal model at lower concentrations (from 10 μM) than MDMA (1 mM), could also indicate that the main mechanism of MDMA toxicity in P19 neurons was not due to its action on SERT. However, tricyclic antidepressants such as clomipramine are also known to interact with other targets including muscarinic acetylcholine receptors [73].

5-HT_2A_ receptors are proposed to be involved in MDMA neurotoxicity. Treatment with ketanserine, a 5-HT_2A_ receptor antagonist was protective against MDMA-induced toxic effects *in vivo* [74] and *in vitro* in cortical neuronal cultures [47]. In the P19 neurons, however, ketanserine did not reduce cytotoxicity produced by MDMA suggesting that 5-HT_2A_ receptors were not involved in the mechanism of toxicity in these cells.

Oxidative stress is another proposed mechanism behind MDMA toxicity [4,5]. Treatments of rodents with antioxidants were neuroprotective against MDMA-induced damage in the striatum [75] and the hippocampus [76]. In the P19 neurons, however, the antioxidants *N*-acetyl-L-cysteine and (+)- α-tocopherol did not decrease the toxic effects of MDMA suggesting that oxidative stress was not the main cytotoxicity cause under the conditions used. The effects of MDMA upon mitochondrial function are involved in cell death produced by MDMA in cultured primary hippocampal neurons from rat embryos [56], and MDMA have been shown to inhibit mitochondrial activity in rodents [8,68]. However, in our P19 neuronal model, MDMA did not significantly alter the mitochondrial membrane potential as measured by the TMRE assay.

In conclusion, MDMA produces toxicity to mouse P19 neurons. Our mechanistic data was essentially negative, but this is important since it rules out a number of possible targets for the toxicity: inhibition of MAO, serotonin re-uptake transporter, 5-HT_2A_ receptor agonism and alternations in mitochondrial membrane potential. Glutamate excitotoxicity has also been implicated in MDMA toxicity *in vivo* [6,7], but this is unlikely to be the case in the P19 neurons, since glutamate produces only mild effects even at high concentrations [32]. Metabolites of MDMA have been suggested to be the major contributors for MDMA neurotoxicity in human cell lines [55], but this has not been addressed in the present study.

## Supporting Information

S1 Fig Concentration-dependent effects of fluoxetine on MDMA-induced toxicity in P19 neurons. P19 neurons, cultured for eight days in the serum-free media, were exposed to fluoxetine for 48 h in presence or absence of 1 mM MDMA. Cell viability was assessed by (A) MTT reduction and (B) LDH activity assay. Data are means ± SEM of n = 5 independent experiments. For MTT reduction assay, data are expressed as percentage of non-treated cells. For LDH release assay, results are presented as percentage of total cell death (cells treated with 2% Triton X-100). Statistically significant differences (using repeated measures one-way ANOVA with post hoc Bonferroni's multiple comparisons test) are indicated: *p < 0.05, ****p < 0.0001, when fluoxetine treatments are compared with corresponding control, and †p < 0.05, ‡p < 0.0001 when MDMA treatments are compared with untreated control cultures.
(TIFF)

S2 Fig Concentration-dependent effects of clomipramine upon viability of P19 neurons. P19 neurons, cultured for seven days in the serum-free media were treated with clomipramine for 24 h at 37°C, in a humified atmosphere with 5% CO_2_. Cell viability was assessed by (A) MTT reduction and (B) LDH activity assay. Data are means ± SEM of n = 3 independent experiments. For MTT reduction assay, data are expressed as percentage of non-treated cells. For LDH release assay, results are presented as percentage of total cell death (cells treated with 2% Triton X-100). Statistical analysis was performed using one-way ANOVA with post hoc Dunnett’s multiple comparisons test (**p< 0.01, ****p< 0.0001) compared to corresponding controls.
(TIFF)
